# The role of tumor‐associated macrophages in hepatocellular carcinoma progression: A narrative review

**DOI:** 10.1002/cam4.6717

**Published:** 2023-12-14

**Authors:** Xinyi Zhang, Chao Yu, Siqi Zhao, Min Wang, Longcheng Shang, Jin Zhou, Yong Ma

**Affiliations:** ^1^ Department of General Surgery, Nanjing First Hospital Nanjing Medical University Nanjing China

**Keywords:** drug resistance, hepatocellular carcinoma, signaling pathway, tumor microenvironment, tumor‐associated macrophages

## Abstract

Hepatocellular carcinoma (HCC) is one of the most common malignant tumors in the world, with complex etiology and mechanism, and a high mortality rate. Tumor‐associated macrophages (TAMs) are an important part of the HCC tumor microenvironment. Studies in recent years have shown that TAMs are involved in multiple stages of HCC and are related to treatment and prognosis in HCC. The specific mechanisms between TAMs and HCC are gradually being revealed. This paper reviews recent advances in the mechanisms associated with TAMs in HCC, concentrating on an overview of effects of TAMs on drug resistance in HCC and the signaling pathways linked with HCC, providing clues for the treatment and prognosis determination of HCC.

## 1 BACKGROUND

Tumor‐associated macrophages (TAMs) are macrophages differentiated from monocytes that are recruited to tumor tissues through chemokines in the tumor microenvironment.^1^ An increasing number of studies have found that TAMs play an important role in a variety of tumors, such as liver cancer,^2^ breast cancer,^3^ gastrointestinal cancer,^4^ ovarian cancer.^5^ Therefore, TAMs have become a hot spot in cancer research in recent years. Among organs in the body, the liver contains the most macrophages after the brain and lung.^6^ TAMs are engaged in liver diseases ranging from acute injury to chronic inflammation, fibrosis and tumor.^7^ It is a worldwide health challenge that liver cancer is expected to affect more than 1 million people annually by 2025.^8^ About 90% of instances of primary liver cancer are hepatocellular carcinoma (HCC), which is also the fourth most common cause of cancer‐related deaths worldwide; HCC is concerned with the interactions of multiple factors, consisting of susceptibility genes, viral and non‐viral risk factors such as fatty liver, immune cells, and tumor microenvironment.^8^ The current treatments for liver cancer include surgical resection, immunotherapy, targeted therapy, transcatheter arterial chemoembolization (TACE), liver transplantation, etc. In terms of drug therapy, drug resistance is still a challenge. Current research has shown that TAMs influence the occurrence, proliferation, invasion, and metastasis of tumors in different ways. With in‐depth studies of the mechanism of TAMs, it is worth looking forward to alleviating drug resistance and improving the effect of monotherapy through regulating TAMs.

## 2 CHARACTERISTICS OF TAMS

Yolk sac, fetal liver, and bone marrow are recognized as three sources of tissue‐resident macrophages differentiated from progenitor cells and monocytes; cancer induces bone marrow generation and mobilization of hematopoietic stem and progenitor cells (HSPC) located in the spleen.^2^, ^9^ Then, monocytes generated in the spleen and bone marrow infiltrate the tumor further and differentiate into TAMs.^2^, ^9^ Macrophages can be classified as M1 and M2 according to phenotype, and markers comprise transmembrane glycoproteins, growth factors, hormones, cytokines, and cytokine receptors. M1, activated by toll‐like receptor (TLR), tumor necrosis factor (TNF)‐α, interferon‐gamma (IFN‐γ), and CSF2, has pro‐inflammatory, anti‐tumor, and bactericidal functions, with markers such as CD86, CD11c, HLA‐DR, iNOS, and pSTAT1.^10^ M2, activated by IL‐4, IL‐10, IL‐13, transforming growth factor (TGF)‐β, and PGE2, plays an important role in anti‐inflammatory, pro‐tumor processes, and tissue repair, with markers such as CD206, CD163, CD204, Arginase‐1, Ym1, MGL‐1, Dectin‐1, vascular endothelial growth factor (VEGF), and cMAF.^10^, ^11^ The infiltration level of M2 polarized macrophages is elevated in patients with hepatocellular carcinoma, and M2 can secrete IL‐6, VEGF, matrix metalloprotease (MMP)‐9, GM‐colony‐stimulating factor (CSF), IL‐10, and monocyte chemoattractant protein‐1 (MCP‐1).^12^ Six macrophage clusters were identified in comprehensive analysis of immune cell composition in HCC patients from five immune‐related sites with full‐length and 3′scRNA‐Seq technology, and mutually exclusive signals of S100 calcium‐binding protein A8 (S100A8) and SLC40A1 were detected on different cells in CD68 macrophages, indicating the existence of two distinct macrophage states in HCC, namely the coexistence of M1 and M2.^13^ M1 and M2 display opposite effects, the polarization of which represents the two extremes of macrophages, and their imbalance is connected to various diseases and inflammations, the M2 phenotype predominant in TAMs in many studies. However, the two are inseparable throughout the cancer process. M1 macrophages generate inflammatory precancerous ecological niches and stimulate early oncogenic mutations, while M2 is reprogrammed to release various growth factors and provide an immunosuppressive state in tumor microenvironment (TME), prompting cancer cells to establish a new vascular system.^14^ Kupffer cells (KCs) are resident macrophages in the liver, considered as specific type of TAMs and secrete relevant cytokines to promote HCC development.^15^, ^16^ There are interactions, consisting of both direct crosstalk and indirect crosstalk via cytokines/chemokines, NK cells, T cells, and neutrophils, between TAMs and hepatocellular carcinoma cells.^17^ More and more studies have demonstrated that TAMs are metabolically heterogeneous and phenotypically plastic, with different impacts on tumor progression and immune function in different contexts.^18^, ^19^

## 3 ROLE OF TAMS IN HCC

### 3.1 Impact of TAMs on the development of HCC

The development of cancer requires a suitable tumor microenvironment that attenuates the killing ability of immune cells. Under hypoxia, triggering receptor expressed on myeloid cells‐1 (TREM‐1) is highly expressed in TAMs, upregulating C‐C motif ligand (CCL)20 via the ERK/nuclear factor kappa B (NF‐κB) pathway and recruiting CD4^+^CD25^+^CCR6^+^Foxp3^+^ Tregs, then inducing CD8^+^T cell apoptosis and dysfunction, thus creating an immunosuppressive environment.^20^ Importantly, blocking programmed death ligand 1 (PD‐L1) does not reverse immunosuppression despite the high expression of PD‐L1 in TREM‐1 TAM, and blocking TREM‐1 signaling is required to reduce resistance to anti‐PD‐L1 therapy.^20^ The expression of cyclooxygenase‐2 (COX‐2) is associated with the M2 macrophage marker CD163/CD206 in tissue chips and paraffin sections of HCC patients and inhibits the synthesis of IFN‐γ and granzyme B+ from activated CD8^+^T cells via the TGF‐β pathway, which results in the loss of CD8^+^T cells' ability to fight tumors.^21^ TAMs also participate in immunosuppression and immune escape by expressing other substances, such as Siglec‐10^22^ and MARCKS,^23^ leading to poor prognosis. In addition to the immunosuppressive environment, cancer cells demand certain nutrients to support growth and proliferation. The number of TAM infiltration and MMP‐9 expression is positively relevant to tumor vascular density, and TAM increases MMP‐9 expression to promote angiogenesis in HCC, involved in type 1 insulin‐like growth factor (IGF‐1) signaling via phosphatidylinositol3‐kinase (PI3‐K) and mitogen‐activated protein kinase (MAPK) pathways.^24^ One study has discovered that zoledronic acid (ZA) impedes TAMs infiltration and secretion of VEGF in a rat HCC model, thereby unfavorable for tumor angiogenesis.^25^

### 3.2 Impact of TAMs on the invasion and metastasis of HCC

Epithelial‐mesenchymal transition (EMT) and cancer stem cell (CSC) are significant pathways for tumor invasion and metastasis. EMT is the conversion of polarized epithelial cancer cells into mesenchymal cells by disassembling adhesion and tight junctions and benefits the separation of mesenchymal cells from the initial site, then passing through the dismantled basement membrane and reaching distant organs, finally returning to the epithelial cancer cell phenotype through mesenchymal‐epithelial transformation and regaining the competence to proliferate and differentiate.^26^ EMT not only expands cell invasion, but also aggravates resistance to cell death, senescence, and therapy and confers stem cell properties on cells.^27^, ^28^ As a driver of tumor invasion and metastasis, CSC can maintain tumor heterogeneity, possess the capability of immune escape, and perform in the formation of immunosuppressive tumor microenvironment.^29^ Activation of EMT and CSC is often influenced by the local microenvironment. TAMs can produce cytokines, induce EMT in HCC cells, and enhance CSC characteristics, thus improving the ability of invasion and metastasis. In the margins of human HCC, there is a positive correlation between the number of TAM and the density of CSC. TAM induces HCC cells to display CSC‐like features, undergo EMT, and acquire more invasion ability by secreting more TGF‐β1, which are linked to a bad prognosis for patients.^30^ TNF‐α can be produced by M2, and through the Wnt/β‐catenin pathway, cancer cells undergo EMT and obtain stemness.^31^ Under hypoxia conditions, TAMs increase IL‐6 secretion and accelerate EMT.^32^ The effects of hypoxia on TAM and HCC are described below. Cancer‐associated fibroblast (CAF), one of the major stromal cells in the HCC tumor microenvironment, encourages the polarization of TAMs into M2 phenotype in vitro simulation experiments while producing CXCL12 to stimulate M2 to secrete plasminogen activator inhibitor‐1 (PAI‐1), which mediates EMT to strengthen the malignant behavior of HCC cells.^33^ The TAM‐mediated angiogenesis described above is important in tumor development, as well as in tumor invasion and metastasis. It not only provides nutrients, but also is one of the good choices of metastasis.

### 3.3 Impact of TAMs on the drug resistance of HCC

Tumor‐associated macrophages are involved in every stage of the development of a tumor, and their infiltration may be used to target cancer prevention or treatment or as a predictive marker for clinical outcomes in a variety of cancers.^34^ Based on numerous research on mechanisms of TAMs affecting HCC, TAMs play an irreplaceable role in HCC, so it is considered meaningful and promising to treat HCC by targeting TAMs. As many drugs targeting TAMs are still in clinical trials at present, main mechanisms are to eliminate the existence of TAMs, block the recruitment of TAMs, reprogram the polarization of TAMs, regulate products of TAMs, and restore the phagocytic ability of TAMs.^35^ TAMs can result in drug resistance in HCC, and hence targeting TAMs may alleviate drug resistance and improve the efficacy of anti‐tumor therapy (Table 1).

**TABLE 1:** Impact of targeting tumor‐associated macrophages (TAMs) on drug resistance in hepatocellular carcinoma (HCC).

Gene/compound/drug	Result	Reference
Adeno‐associated virus 8	Mediate hepatic IRF8 rescue, inhibiting TAMs infiltration and decreasing expression of CCL20, and significantly inhibit HCC progression, enhancing the response to anti‐PD‐1 therapy	^36^
Carbonic anhydrase XII (CA12) inhibitor	Reduce TAM infiltration and CCL8 production and attenuate tumor growth and metastasis, promoting anti‐PD1 therapy	^37^
Celecoxib	Downregulate the expression of Foxp3Tregs, CD68 TAM, and PD‐L1 and increase the infiltration of CD8 CTL, augmenting the therapeutic efficacy of epirubicin	^38^
Competitive binding antibody Siglec‐10 Fc	Block Siglec‐10 expressed by TAM, decrease expression of immunosuppressive molecules, and increase the cytotoxic effects of CD8^+^ T cells against HCC cells, leading to promoting the anti‐tumor efficacy of the PD‐1 inhibitor pembrolizumab	^22^
COX‐2 inhibitor (celecoxib)	Reduce the inhibitory effect on CD8^+^ T cells through regulating TAMs in TIME, thus enhancing the efficacy of T cell‐based immunotherapy	^21^
CSF1/CSF1R inhibitor PLX3397 (PFH@LSLP)	Activate the immune responses via inhibiting the CSF1/CSF1R pathway in TAMs, further enhance CD8^+^ T cell infiltration to reverse immunosuppression in tumors, thus overcoming sorafenib resistance	^39^
CSF1R inhibitor BZL945/IL‐1R1 antagonist anakinra	Attenuate SLC7A11‐mediated intratumoral TAM and MDSC infiltration, enhancing the immune response to anti‐PD‐L1 therapy	^40^
IFN‐α	Increase sorafenib's therapeutic efficacy by shifting TAM polarization to an M1‐like phenotype, increasing and activating intratumoral CD8^+^ T cells in HCC	^41^
Inhibition of APOC1	Promote the transformation of M2 into M1 via the ferroptosis pathway, thereby reshaping the tumor immune microenvironment, resulting in enhancing sensitivity to anti‐PD1 therapy in HCC	^42^
Lmdd‐MPFG (Listeria‐based HCC vaccine)	Promote anti‐PD‐1 therapy through skewing the TAMs from M2 into M1	^43^
MTL‐CEBPA (small activating RNA)	Reverse the immunosuppressive activity of M‐MDSCs and TAMs, promoting the anti‐tumor effect of checkpoint inhibitors or PMN‐MDSC‐targeted immunotherapy	^44^
RNA interference of autophagy‐related 5 homolog (ATG5)	Suppress autophagy activated by co‐culturing with macrophages in HCC cells, promoting the oxaliplatin cytotoxicity	^45^
Sorafenib	Upregulate IL‐12 production in TAMs at a sub‐pharmacologic, increasing the anti‐tumor effect of mCART cells therapy	^46^
Triggering receptor expressed on myeloid cells‐1 (TREM‐1) inhibitor GF9	Abrogate immunosuppression mediated by TREM‐1TAM, strengthening the effect of anti‐PD‐1 therapy	^20^
TREM2 knockdown	Remodel TAMs to an immune‐stimulating status greatly improve the therapeutic effect of PD‐1 blockade probably through increasing the infiltration of immune cells and enhancing the toxicity of infiltrated CD8, CD4, and NK cells	^47^
xCT knockout	Mediate ferroptosis to significantly increase PD‐L1 expression in macrophages and improve the anti‐tumor efficacy of anti‐PD‐L1 therapy	^48^
Zoledronic acid (ZA)	Enhance the effects of transcatheter arterial chemoembolization through inhibiting TAM infiltration and tumor angiogenesis in rat HCC models	^25^
	Reduce PD‐L1^+^ TAMs infiltration and alleviate CD8^+^ T cell suppression, enhancing the efficacy of anti‐PD‐L1 therapy in HCC	^49^

Sorafenib, used in the first‐line systemic therapy approved by the U.S. Food and Drug Administration (FDA) and the standard of care for advanced hepatocellular carcinoma, is affected by different factors in many studies, and the percentage of beneficiaries is unable to reach a satisfactory standard with decreased sensitivity and increased resistance to sorafenib in HCC patients.^50^ Accordingly, it is particularly vital to take combination therapy with other inhibitors. M2 TAMs offer hepatocyte growth factor (HGF) to activate the HGF/c‐Met, ERK1/2/MAPK, and PI3K/AKT pathways, enlisting more macrophages to tumor tissue and intensifying HCC resistance to sorafenib in a pre‐feedback manner.^51^ Studies have presented that targeting TAMs probably overcomes resistance to sorafenib and raises therapeutic efficacy. In H22 and PDX mice model of HCC, PLX3397 further enhances CD8 T‐cell infiltration and activates intracellular immune responses by inhibiting the CSF1/CSF1R pathway in TAMs and reducing TAMs recruitment and M2 polarization, which is more effective and comprehensive to conquer sorafenib resistance in synergy with alleviating hypoxia.^39^ IFN‐α triggers the transition from M2 to M1 to neutralize the proliferative and migratory effects on HCC, simultaneously expanding CD8^+^T‐cell infiltration in HCC and enhancing the therapeutic effect of sorafenib.^41^ Furthermore, sorafenib acts on TAMs in turn. Sorafenib augments the anti‐tumor effect of mouse chimeric antigen receptor (mCAR) T‐cell therapy through stepping up IL‐12 produced by TAMs at a sub‐pharmacologic dose.^46^

The ascent of PD‐L1 is a critical mechanism of tumor immune escape. TAMs are able to express PD‐L1, and regulation of TAMs is significantly meaningful for PD‐1/PD‐L1 immunotherapy. CA12 inhibitor dwindles TAMs infiltration and CCL8 production, attenuating tumor growth and metastasis with growing proportion of CD8^+^T cells, thus showing more notable efficacy in combination with anti‐PD‐1 therapy than monotherapy.^37^ Lmdd‐MPFG, a Listeria‐based HCC vaccine, promotes PD‐L1 expression in HCC cells but restores tumor local T‐cell sensitivity and gains the response to anti‐PD‐1 therapy by switching TAMs from M2 to M1 polarization.^43^ Zoledronic acid restrains PD‐L1 TAM infiltration and attenuates CD8 T‐cell suppression, then enhancing the effect of anti‐PD‐L1 therapy in hepatocytes, especially in patients with Golgi membrane protein 1 (GOLM1) overexpression.^49^ CSF1R inhibitor BZL945^40^ and TREM‐1 inhibitor GF9^20^ facilitate anti‐tumor curative effect of anti‐PD‐L1 in HCC as well.

Moreover, oxaliplatin,^45^ epirubicin,^38^ TACE,^25^ and other relevant treatments for liver cancer may be more efficient by modulation of TAMs. The above results suggest that the regulation of TAMs will reverse the immunosuppressive environment and regain the position of T cells in the immune response. Compared with single therapy, combination therapy is a direction in the treatment of HCC, worthy of consideration and application.

## 4 SIGNALING PATHWAY/AXIS INVOLVED IN HCC VIA TAMS

**FIGURE 1:**
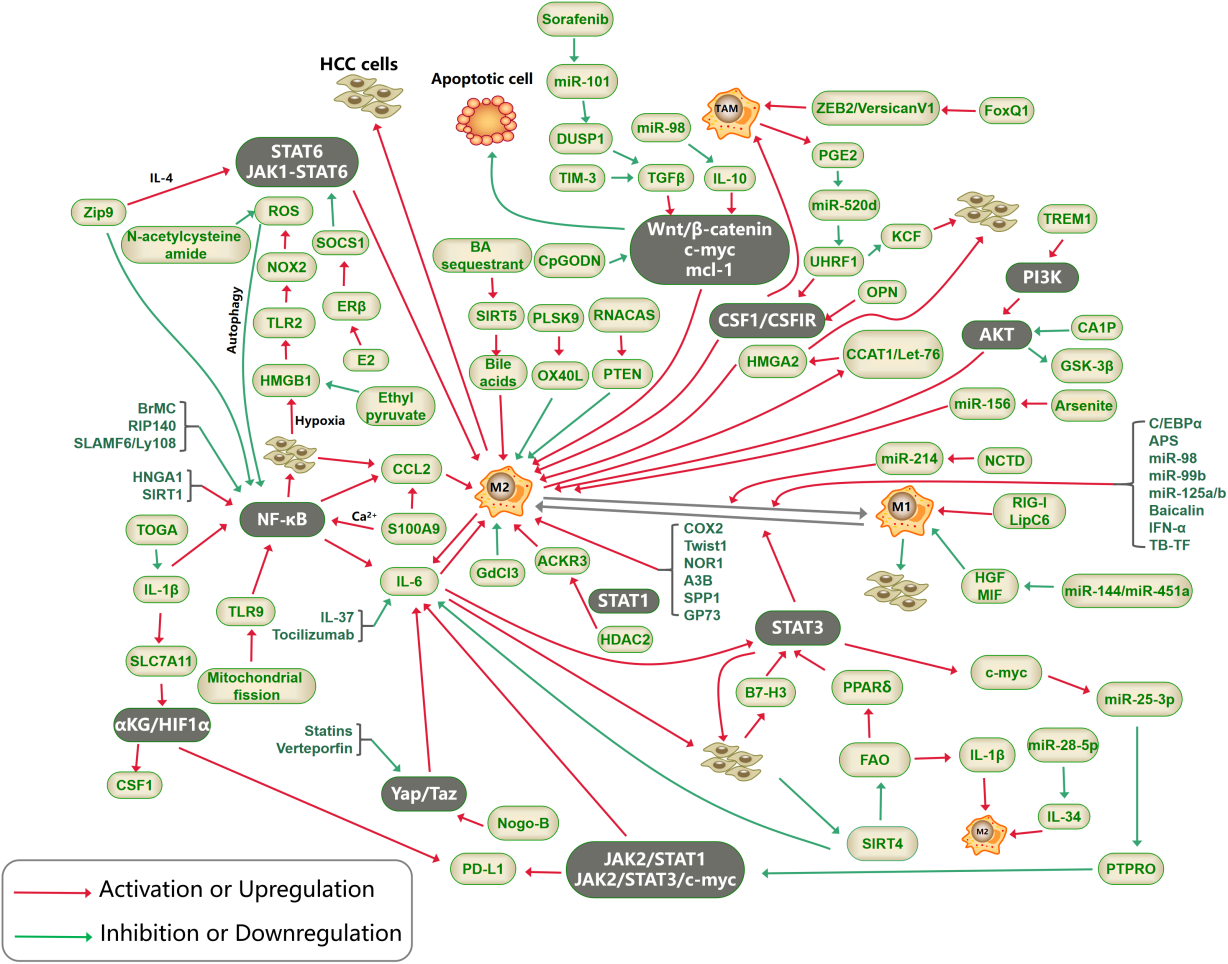
The signaling pathways associated with tumor‐associated macrophages (TAMs) in hepatocellular carcinoma (HCC).

In recent years, a growing number of studies have found that TAMs are considered to occupy an irreplaceable position in the pathogenesis of HCC, including immunosuppression, angiogenesis, tumor invasion and metastasis, metabolic support, drug resistance, EMT, and malignant transformation of HCC stem cells.^35^, ^52^ TAMs are either active to function on HCC or stimulated by factors secreted from hepatocellular carcinoma cells or foreign factors to act on tumor tissue (Table 2 and Table 3), bringing about the promotion or inhibition of HCC progression by means of recruitment, infiltration, polarization, secretion of chemokines, and autophagy. Multiple signaling pathways/axes play a key role in the crosstalk between TAMs and HCC (Figure 1).

**TABLE 2:** Impact of regulators on tumor‐associated macrophages (TAMs) in hepatocellular carcinoma (HCC).

Gene/compound/drug/axis	Mechanism	Influence on TAMs	Influence on HCC	Reference
APOBEC3B (A3B)	Depress global H3K27me3 abundance via interaction with PRC2 and reduces an occupancy of H3K27me3 on promoters of the chemokine CCL2 to recruit massive TAMs and MDSCs	Promote M2	+	^53^
Astragalus polysaccharin (APS)	Repress M2 polarization of TAMs	Promote M1	−	^54^
		Inhibit M2		
Arsenite	Increase miR‐15b levels and induce M2 polarization of THP‐1 cells	Promote M2	+	^55^
B7 homolog 3 (B7‐H3)	Mediate STAT3 signaling pathway to induce M2‐type polarization of TAMs	Promote M2	+	^56^
Baicalin	Autophagy‐induced RelB/p52 activation mediates repolarization of TAM to M1‐like phenotype	M2 → M1	−	^57^
Blocking of the CCL2/CCR2 axis	Inhibit the recruitment of inflammatory monocytes, infiltration, and M2‐polarization of TAMs	Inhibit M2	−	^58^
8‐bromo‐7‐methoxychrysin (BrMC)	Reverse M2 polarization of TAMs due to inhibition of NF‐κB activation	Reverse M2	−	^59^
CCAAT/enhancer‐binding protein alpha (C/EBPα)	Reverse the immunosuppressive activity of M‐MDSCs and TAMs	M2 → M1	−	^44^
Combretastatin A‐1 phosphate (CA1P)	Induce TAM apoptosis in vitro and eliminate TAMs in the TME in vivo through GSK‐3β activation, Wnt/β‐catenin pathway inhibition and Mcl‐1 downregulation	Eliminate TAM	−	^60^
Cancer‐associated fibroblasts (CAFs)	Induce the M2 polarization of TAMs by upregulating the mRNA expression levels of CD163 and CD206 and downregulating IL‐6 mRNA expression and secretion in the macrophages, and induce PAI_x005f‐1 secretion via CXCL12	Promote M2	+	^33^
Cholestyramine	Reverse the effect of Sirt5 deficiency in promoting M2‐like polarized TAMs and liver tumor growth	Inhibit M2	−	^61^
Cyclooxygenase‐2 (COX‐2)	Induce anti‐tumor abilities exhaustion in activated CD8+ T cell through M2 TAMs polarization and transforming growth factor (TGF) beta pathway	Promote M2	+	^21^
DNaseI/TLR9 antagonist	By depleting cytosolic mtDNA or blocking TLR9 pathway, respectively, siRNA for TLR9 or p65 in HCC cells with Drp1 overexpression significantly decrease the recruitment and polarization of TAMs	Inhibit M2	−	^62^
Downregulation of TREM1	Shift M2 macrophages toward a M1 phenotype via inhibiting PI3K/AKT signaling	M2 → M1	−	^63^
17β‐estradiol (E2)	Function as a suppressor for macrophage alternative activation and tumor progression by keeping ERβ away from interacting with ATP5J, thus inhibiting the JAK1‐STAT6 signaling pathway	Inhibit M2	−	^64^
Ethyl pyruvate and N‐acetylcysteine amide	Block HMGB1 and ROS respectively, significantly reducing M2 macrophage recruitment	Inhibit M2	−	^65^
Fatty acid oxidation (FAO)	Contributes to IL‐1β secretion in M2 macrophages and the pro‐migratory effect in M2 MDMs	Promote M2	+	^66^
Forkhead box Q1 (FoxQ1)	Transactivate ZEB2 and VersicanV1 expression, resulting in the induction of EMT and the recruitment of macrophage infiltration	Promote recruitment	+	^67^
Gadolinium chloride (GdCl3)	Downregulate the expression of CD206 in TAMs	Inhibit M2	−	^68^
Genipin	Suppress IRE1α‐mediated infiltration and priming of TAMs	Inhibit infiltration	−	^69^
Granulocyte‐macrophage colony‐stimulating factor (GM‐CSF)	Enhance A2A receptor expression on Mϕ and function synergistically with adenosine to elicit Mϕ proliferation	Promote M2	+	^70^
Golgi membrane protein 1 (GOLM1)	Induces CD8+ T cells suppression through promoting PD‐L1 stabilization and transporting PD‐L1 into TAMs with exosome dependent	Promote M2	+	^49^
Golgi protein 73 (GP73)	Stimulate endoplasmic reticulum (ER) stress activation in neighboring macrophages, which then release cytokines and chemokines involved in the TAM phenotype	Promote M2	+	^71^
Histone deacetylase 2 (HDAC2)	Upregulate atypical chemokine receptor 3 (ACKR3) via STAT1 to induce migration of M2 macrophages and immune escape in HCC	Promote M2	+	^72^
Hemozoin (HZ)	Inhibit IGF‐1 signaling through the PI3‐K and MAPK signaling pathways and thereby decrease the expression of MMP‐9 in TAMs, suppressing tumor angiogenesis	Inhibit M2	−	^24^
High‐mobility group A1 (HMGA1)	Promote macrophage recruitment by activating NF‐κB‐CCL2 signaling	Promote recruitment	+	^73^
HMGA2	Promote the migrating abilities of both M0‐Mφs and TAMs‐Mφs	Promote M2	+	^74^
High‐mobility group box 1 (HMGB1)	Produced by hepatoma cells in a HIF‐1α‐dependent manner under hypoxia, inducing the infiltration and reprogramming of macrophages to augment the expression of IL‐6	Promote M2	+	^32^
	Trigger M2 macrophage polarization through HMGB1/TLR2/NOX2/autophagy axis	Promote M2	+	^65^
Heat shock transcription factor 1 (HSF1)	Regulate individually monocarboxylate transporter 1 (MCT1) and MCT4 expressions not only in HCC cells but also in TAMs, decreasing glucose consumption rate, lactate production rate and intercellular ROS level	Promote M2	+	^75^
Interferon gamma (IFNG)	Induce M2 polarization and chemotactic migration of macrophages through IFNG/IFR1/HHLA2 axis in HCC	Promote M2	+	^76^
IFN‐α	Shift TAM polarization to an M1‐like phenotype, increase and activate intratumoral CD8+ T cells in HCCs	M2 → M1	−	^41^
IL‐1β	Induce SLC7A11 overexpression to upregulate PD‐L1 and CSF1 through the αKG/HIF1α axis, promoting TAM and MDSC infiltration	Promote recruitment	+	^40^
IL‐2	Modulate exosomal miRNAs from TAMs to ameliorate hepatocellular carcinoma development	−	−	^77^
IL‐37	Promote TAMs polarization from M2 to M1 subtype through inhibiting the IL‐6/STAT3 signaling	M2 → M1	−	^78^
IL‐6	Decrease PTPRO expression through the STAT3/c‐MYC/miR‐25‐3 p axis, promoting PD‐L1 secretion in both monocytes and macrophages	Promote M2	+	^79^
Interleukin‐1 receptor‐associated kinase 1 (IRAK1) silencing	Reverse the TAM‐induced increase in expression of NLRP3 and pro‐inflammatory factors in HCC cells	Inhibit M2	−	^80^
Interferon regulatory factor 8 (IRF8)	Mediate repression of c‐fos transcription resulting in decreased expression of CCL20 to regulate recruitment of TAMs	−	−	^36^
Nanoliposome‐loaded C6‐ceremide (LipC6)	Reduce the number of TAMs and the ability of TAMs to suppress the anti‐tumor immune response and induce TAMs to differentiate into an M1 phenotype	Promote M1	−	^81^
Long non‐coding RNA GAS5	Inhibit M2‐like polarization of TAMs by enhancing PTEN expression	Inhibit M2	−	^82^
Myristoylated alanine‐rich C‐kinase substrate (MARCKS)	Influence the M2 polarization and immune escape	Promote M2	+	^23^
Mesoporous Fe3O4 nanoparticles (mFe NPs)	Reprogram TAMs into M1 phenotypes to synergistically amplify anti‐tumor immunity after TAM phagocytosis	Promote M1	−	^83^
miR‐125a/b	Inhibit tumor‐associated macrophages mediated in cancer stem cells of hepatocellular carcinoma by targeting CD90	Inhibit M2	−	^84^
miR‐144/miR‐451a cluster	Promote macrophage M1 polarization and anti‐tumor activity by targeting HGF and MIF	Promote M1	−	^85^
miR‐28‐5p deficiency	Upregulate IL‐34 and promote TAM infiltration	Promote M2	+	^86^
miR‐98	Regulate expression of inflammatory cytokines in HCC‐conditioned TAMs and modulated the capacity of HCC‐conditioned TAMs to regulate HepG2 cell migration and invasion by targeting IL‐10	Inhibit M2	−	^87^
	Modulate macrophage polarization from M2 to M1	M2 → M1	−	^88^
miR‐99b	Promote M1 while suppressing M2 macrophage polarization by targeting κB‐Ras2 and/or mTOR respectively	Promote M1	−	^89^
		Inhibit M2		
Mitochondrial fission	Induce the cytosolic mtDNA stress to enhance the CCL2 secretion from HCC cells by TLR9‐mediated NF‐κB signaling pathway, and thus promote the TAM recruitment and polarization	Promote M2	+	^62^
MYC and Twist1	Elicit a transcriptional program associated with the activation of innate immunity through secretion of a cytokinome that elicits recruitment and polarization of TAMs	Promote M2	+	^90^
Norcantharidin (NCTD)	Modulate a shift from M2 to M1 polarization through miR‐214	M2 → M1	−	^91^
Nogo‐B	Promote TAMs M2 polarization by inducing Yap/Taz pathway	Promote M2	+	^92^
NOTCH blockade	Impede the differentiation of moTAMs, but upregulates Wnt/β‐catenin signaling to promote the proliferation and pro‐tumor cytokine production of kclTAMs	Inhibit moTAM	+	^16^
		Promote kclTAM		
Osteopontin (OPN)	Induce chemotactic migration and M2‐like polarization of macrophages and promote the PD‐L1 expression in HCC via activation of the CSF1‐CSF1R pathway in macrophages	Promote M2	+	^93^
Proprotein convertase subtilisin/kexin type 9 (PCSK9)	Inhibit M2 polarization of TAMs in HCC by promoting OX40L expression	Inhibit M2	−	^94^
Propofol	Stimulate TAMs to secrete microvesicles, mediating transfer of miR‐142‐3p from macrophages to cancer cells in vivo	Promote M1	−	^95^
		Inhibit M2		
Retinoic acid‐inducible gene I (RIG‐I)	Promote the polarization of M1 through the RIG‐I/MAVS/TRAF2/NF‐κB pathway in mice peritoneal macrophages	Promote M1	−	^96^
Receptor‐interacting protein 140 (RIP140)	Inhibit M2 polarization and the activation of NF‐κB/IL‐6 axis in the TAMs	Inhibit M2	−	^97^
	Inhibit the alternative activation of macrophages by inhibiting the NF‐κB/IL‐6 axis in TAMs	Promote M1	−	^98^
		Inhibit M2		
Receptor‐interacting protein kinase 3 (RIPK3) deficiency	Reduce reactive oxygen species and significantly inhibit caspase1‐mediated cleavage of PPAR, facilitating fatty acid metabolism and inducing M2 polarization in the tumor microenvironment	Promote M2	+	^99^
S100 calcium‐binding protein A9 (S100A9)	Recruit more macrophages via CCL2	Promote M2	+	^100^
Semaphorin3A	Increase TAM infiltration	Promote M2	+	^101^
Sirtuin 1 (SIRT1)	Enhance NF‐κB stimulation, promoting phosphorylation of p65, IκB, and IκB kinase, to reinforce M1‐like macrophage infiltration	Promote M1	−	^102^
SIRT4 silencing	Facilitate M2 polarization via the FAO‐PPARδ‐STAT3 axis	Promote M2	+	^103^
SIRT5 deficiency	Elevate bile acids abnormally and promote M2‐like macrophage polarization	Promote M2	+	^61^
Signaling lymphocyte activation molecule family member 6 (SLAMF6/Ly108)	Promote macrophage M2 polarization	Promote M2	+	^104^
Sorafenib	Inhibit miR‐101 expression, enhance DUSP1 expression and lower TGF‐β and CD206 release in M2 cells	Inhibit M2	−	^105^
SPP1	Trigger the polarization of macrophages to M2‐phenotype TAMs via SPP1‐CD44 and SPP1‐PTGER4 association	Promote M2	+	^106^
Statins (inhibitors of hydroxymethylglutaryl‐CoA reductase)	Inhibit YAP‐induced IL‐6 expression to block the recruitment of TAMs	Inhibit M2	−	^107^
Tannin fraction of Terminalia bellirica (TB‐TF)	Inhibit orthotopic tumor growth and promote the polarization of M2‐TAMs toward the anti‐tumor M1 phenotype in vivo	M2 → M1	−	^108^
T cell immunoglobulin and mucin‐domain containing protein‐3 (Tim‐3)	Enhance TGF‐β‐mediated alternative activation of macrophages	Promote M2	+	^109^
TLR9 agonist CpGODN	Downregulate the level of glycolysis and inhibit the M2 polarization of HCC‐TAMs	Inhibit M2	−	^110^
Tocilizumab	Block IL6 signaling to inhibit TAM‐stimulated activity of CD44 (+) cells	Inhibit M2	−	^111^
Glycyrrhetinic acid‐tetramethylpyrazine conjugate (TOGA)	Inhibit IL‐1β‐induced activation of the IL‐1R1/IκB/IKK/NF‐κB signaling pathway, effectively preventing the support of TAMs from fueling tumorigenesis	Inhibit M2	−	^112^
Ubiquitin‐like with PHD and ring finger domains 1 (UHRF1)	Induce DNA hypomethylation of the CSF1 promoter, promoting CSF1 expression, thereby leading to TAM recruitment and activation	Promote recruitment/activation	+	^113^
Verteporfin	Block Nogo‐B‐Yap/Taz‐mediated macrophages M2 polarization	Inhibit M2	−	^92^
Wnt/β‐catenin	Stimulate M2‐like polarization of TAMs	Promote M2	+	^114^
Wnt2b	Promote the polarization of TAMs to M2‐like macrophages by activating Wnt2b/β‐catenin/c‐Myc signaling	Promote M2	+	^110^
Yes‐associated protein (YAP)	Induce IL‐6 secretion, promoting the TAMs recruitment	Promote M2	+	^107^
ZA	Inhibit infiltration of TAMs and tumor angiogenesis	Inhibit M2	−	^25^
Zip9	Enhance phosphorylated STAT6 to promote M2 macrophage polarization but suppress the phosphorylation of IκBα/β to inhibit M1 macrophage polarization	Promote M2	+	^115^
		Inhibit M1		

**TABLE 3:** Impact of tumor‐associated macrophages (TAMs) on hepatocellular carcinoma (HCC).

Gene/compound	Mechanism	Influence on HCC	Reference
xCT	Derived from TAM, facilitates carcinogenesis though increasing TAM recruitment and infiltration, modulating M2 polarization and reducing ferroptosis	+	^48^
CXCL1 and CXCL2	Secreted by M2 TAM, increasing HCC CSC activity, decreasing SOR‐induced apoptosis by affecting BCL‐2 family gene expression and upregulating SOR resistance in HCC cells via CXCR2/ERK	+	^116^
Dectin3	Expressed by macrophages, contributing to the apoptosis of tumor cells and inhibiting the proliferation of tumor cells by regulating the glycolysis of macrophages	−	^117^
Deletion of IL‐6	Inhibit IL‐6/signal transducer and activator of transcription 3 signaling in monocytes/KCs	−	^15^
Gal‐1	Secreted by TAM via secretory autophagy	+	^118^
GM‐CSF	Secreted by TAM, enhancing A2A receptor expression on Mϕ and functioning synergistically with adenosine to elicit Mϕ proliferation in HCC	+	^70^
IL‐6	Produced by TAM, promoting expansion of these CSCs and tumorigenesis via STAT3 signaling	+	^111^
	Produced by TAM, promoting EMT of HCC cells and enhancing the invasiveness and metastasis of murine HCC cells under hypoxia	+	^32^
Inhibition of APOC1	Expressed by TAM, promoting the transformation of M2 macrophages into M1 macrophages via the ferroptosis pathway, thereby reshaping the tumor immune microenvironment and inhibiting HCC progression	−	^42^
lncMMPA	Transmitted by exosome derived from TAM, interacting with miR‐548 s and increasing the mRNA level of ALDH1A3, then further promoting glucose metabolism and cell proliferation in HCC; polarize M2 macrophage	+	^119^
lncRNAH19	Induced by TAM, triggering and activating the miR‐193b/MAPK1 axis	+	^120^
microRNA (miR)‐17–92 cluster	Originating from the EVs of M2‐TAMs, stimulating the imbalance of TGF‐β1/BMP‐7 pathways in HCC cells and increasing inhibitor of differentiation 1 (ID1) expression	+	^121^
miR‐628‐5p	Transferred by exosomes derived from M1 macrophages to HCC cells to inhibit human methyltransferase‐like 14 (METTL14) expression, hindering the m6A modification of circFUT8 competitively binding to miR‐552‐3p to increase CHMP4B expression	−	^122^
NOR1	Expressed by TAM, promote M2 alternative polarization	+	^123^
PGE2	Derived from TAM, stimulating UHRF1 expression by repressing miR‐520d that targets the 3′‐UTR of UHRF1 mRNA	+	^113^
S100A9	Derived from TAM, enhances stem cell traits of HCC cells via AGER/NF‐κB axis	+	^100^
Siglec‐10	Expressed by TAM, exerting immunosuppressive function during HCC progression	+	^22^
TGF‐β1	Produced by TAM, promoting CSC‐like properties via inducing EMT	+	^30^
Tim‐3	Expressed by TAM, promoting tumor growth via NF‐κB‐IL‐6 axis	+	^109^
TLR4	M2‐polarized macrophages facilitate the migration and EMT of HCC cells via the TLR4/STAT3 signaling pathway	+	^124^
Tumor necrosis factor‐α	Derived from M2‐TAMs, promoting EMT and cancer stemness cells via the Wnt/β‐catenin pathway	+	^31^
TREM‐1	Expressed by TAM under hypoxia, elevating CCL20 expression through the extracellular signal‐regulated kinase/nuclear factor kappa B pathway and initiating the onset of tumor immunosuppression through attracting CCR6+ Foxp3+ Tregs	+	^20^
TREM2 knockdown	Expressed by TAM, remodeling TAMs to an immune‐stimulating status and suppressing the growth of hepatocellular carcinoma	−	^47^
β2‐AR	Expressed by TAM, downregulating GRK2 and activating the downstream cyclic adenosine monophosphate (cAMP)/protein kinase A/cAMP‐response element binding protein and cAMP/interleukin‐6/signal transducer and the activator of transcription 3 signaling pathways	+	^12^

### 4.1 STAT signaling pathway

Signal transducers and activators of transcription (STAT) function complexly and importantly in controlling normal physiological cellular processes such as angiogenesis, differentiation, proliferation, apoptosis, and immune system, and meanwhile are used for the epigenetic makeup of immune cells. At the same time, abnormal STAT management may occur, giving rise to pathological events about cancer‐containing occurrence, progression, metastasis, survival, and treatment resistance. To date, seven STAT genes have been identified in the human genome, and among them, STAT3 and STAT5 may be more important in cancer development. STAT3 is the most studied gene and is closely associated with tumor growth and immune escape in most tumors, while STAT5 is mainly in hematologic tumors. STAT1 is considered as a tumor suppressor, linked with the M1 phenotype,^125^, ^126^ whereas STAT3 is thought as a tumor promoter. Currently, it is primarily STAT3 and STAT6 that act between HCC and TAMs according to lots of research.

IL‐6 is a classic inflammatory factor and the most decisive activator of STAT3 that has a core role in transcription factors driving IL‐6‐induced transcriptome alterations in macrophages.^127^ The experiments on patients and xenograft mice demonstrate that TAMs secrete IL‐6, which provokes CSC in HCC through STAT3 signaling.^111^ IL‐6 upgrades miR‐25‐3p through STAT3/c‐MYC signaling to downgrade PTPRO in HCC monocytes, ascending PD‐L1 expression and tumor growth in vivo.^79^ IL‐37 hinders IL‐6/STAT3 signaling to promote polarization of TAMs from M2 to M1 subtypes, constricting proliferation, migration, and invasion of HCC cells.^78^ In Mdr2‐deficient mice that spontaneously develop HCC, loss of IL‐6 in monocytes/KCs contributes to suppression of IL‐6/STAT3 signaling and delayed tumorigenesis.^15^ In addition, there are other factors influencing the role of STAT3 between HCC and TAMs. B7‐H3, a co‐stimulatory molecule involved in the regulation of non‐immune functions, favors PMA‐induced differentiation of THP‐1 cells to the M2 phenotype when overexpressing in HCC, and partial blockade of the STAT3 signaling pathway may inhibit the elevation of B7‐H3 expression on THP‐1 cells.^56^ SIRT4 silencing facilitates M2 TAM polarization through the fatty acid oxidation (FAO)‐PPARδ‐STAT3 signaling pathway.^103^ Toll‐like receptor 4 (TLR4) is a molecular biomarker of aggressive tumors and unfavorable prognoses. Human HCC cells undergo migration and EMT when M2‐polarized macrophages stimulate the STAT3 signaling pathway downstream of TLR4 and amplify TLR4 expression in HCC cells.^124^

The JAK/STAT pathway is a critical pathway to mediate inflammatory response and tumorigenesis. Expression of PTPRO suppresses PD‐L1 expression in HCC macrophages or monocytes through JAK2/STAT1 and JAK2/STAT3/c‐MYC activation.^79^ In a BALB/c mouse ectopic tumor model, E2 is found to restrict the Jak1‐Stat6 signaling pathway by keeping estrogen receptor beta (ERβ) away from interaction with ATPase‐coupling factor 6 (ATP5J), thus acting as an suppressor of macrophage alternative activation and tumor progression, whereas androgen has no significant role in HCC progression.^64^ Furthermore, STAT6 signaling is crucial for invasion of EMT and CRC cells induced by IL‐4 and IL‐13.^128^ Zip9 decreases phosphorylation of the IκBα/β pathway to inhibit M1 polarization and simultaneously increases phosphorylation of STAT6 to stimulate M2 polarization.^115^ Exosome‐mediated ASO suppresses STAT6 expression in TAM and causes effective reprogramming of TAMs to the M1 phenotype in CT26 and Hepa1‐6 tumor model.^129^ Basic leucine zipper ATF‐like transcription factor (BATF), involved in the synergistic induction of target gene expression, is further induced by co‐binding of STAT3 and STAT6, and high levels of BATF expressed from macrophages may contribute to tumor progression.^127^ Consequently, targeting STAT3 and STAT6 pathways is an option for the treatment of HCC.

### 4.2 NF‐κB signaling pathway

NF‐κB is an important nuclear transcription factor. The NF‐κB family consists of five proteins, including RelA, c‐Rel, RelB, p50, and p52, which assemble into multiple homodimers and heterodimers, each with unique function in regulation of transcription in immune system cells and many other cell types.^130^ It not only plays a specific part in inflammation, innate immunity, cancer, and apoptosis,^131^, ^132^ but also promotes hepatocarcinogenesis through liver inflammation, hepatocyte death, and compensatory proliferation.^133^ NF‐κB activation is linked to the induction of carcinogenesis in a number of experimental types of inflammation‐associated malignancies, as it is a fundamental regulator of inflammation, particularly in TAM.^134^ Through the study of HCC peritoneal tissue, the downregulation of SIRT4 can activate the NF‐κB pathway, leading to the downstream upregulation of MCP‐1 gene expression and increasing the infiltration of TAMs.^103^ High‐mobility group A1 (HMGA1) elevates CCL2 expression in an NF‐κB‐dependent manner and induces the recruitment of macrophages in HCC.^73^ Through the TLR9‐mediated NF‐κB signaling pathway, mitochondrial fission causes cytosolic mtDNA stress and increases CCL2 release in HCC cells, which in turn causes TAM recruitment and polarization.^62^ RIP140 overexpression inhibits NF‐κB/IL‐6 axis activation, thereby suppressing M2 polarization to hinder hepatocellular carcinoma cell growth and proliferation.^97^, ^98^ S100A9 enhances the stem cell traits of HCC cells through the AGER/NF‐κB axis,^100^ and Tim‐3 adds IL‐6 production by activating NF‐κB in macrophages, thereby promoting the growth of liver cancer cells.^109^ Experiments have been conducted to reduce expression/activation of this transcription factor with different pharmacological approaches, thus restoring chemosensitivity.^135^ Accordingly, transcription factors can be considered as effective drug targets for oncological diseases. However, the function of NF‐κB activation in TAM may differ based on the tumor microenvironment and tumor development stage. On the one hand, NF‐κB makes for cancer, and on the other hand, it hinders cancer.^132^ TAMs frequently exhibit an anti‐inflammatory phenotype associated with immunosuppression that does not necessarily coincide with the pro‐inflammatory function of NF‐κB in TAMs.^134^ Sirtuin 1 (SIRT1) enhances stimulation of NF‐κB pathway in macrophages and promotes TAM polarization to M1 tumor suppressor phenotype.^102^ Through a positive feedback regulatory loop, MiR‐99b may enhance M1 macrophage function via NF‐κB, leading to elevated antigen presentation and phagocytosis while attenuating M2 polarization.^89^ Due to the contradiction of NF‐κB between cancer inhibition and promotion, the complexity of the whole disease process should be taken into account, which means treatment strategies should be formulated carefully to avoid opposite effects.

### 4.3 Wnt signaling pathway

Hepatocytes that receive Wnt/β‐catenin signaling from the microenvironment have high tumor potential, and the activation of this signaling pathway plays an important role throughout liver regeneration and hepatocarcinogenesis during chronic liver injury.^136^, ^137^ Wnt/β‐catenin signaling pathway can also affect HCC through different impacts on TAMs. After THP‐1‐derived macrophages (THP‐1‐M) are incubated with 50% HCC‐TCM, M2‐type macrophage markers CD163, IL‐10, and CCR2 are upregulated, and further studies reveal that HCC‐TCM promotes M2 polarization through Wnt2b/β‐catenin signaling, inducing HCC to undergo EMT facilitating proliferation and migration.^110^ Orthotopically inoculated hepatic Hepa1‐6 tumors in mice are accelerated by myeloid‐specific NOTCH blockade by conditional disruption of recombination signal binding protein Jκ (RBPj cKO), and NOTCH signaling is negatively correlated with WNT activation in CD68+ macrophages in patient‐derived HCC biopsies, while positively relevant to advanced HCC stages.^16^ Consequently, NOTCH inhibition prevents moTAM differentiation while elevating Wnt/β‐catenin signaling to encourage kclTAM proliferation and pro‐tumor cytokine release, which speeds up the development of HCC and colorectal cancer's liver metastasis.^16^ Besides, CA1P‐induced microtubule depolymerization mediates AKT inactivation, then activates GSK‐3β and downregulates Wnt/β‐Catenin signaling pathway and Mcl‐1, leading to the apoptosis of HepG2 cells; by the same mechanism, TAM apoptosis is induced, and secondary metastasis of TAMs partially rescues the growth of CA1P‐suppressed tumors.^60^ In SMMC‐7721 hepatocellular carcinoma cells and nude mice subcutaneous tumor models, TNF‐α produced by TAMs promotes EMT and CSC via Wnt/β‐catenin pathway.^31^ Another study finds that nuclear accumulation of β‐catenin is positively correlated with CD68 TAM in biopsies from HCC patients; in addition to β‐catenin, levels of Axin2 and c‐Myc are increased in M2, and Wnt/β‐catenin activation stimulates M2 macrophage polarization via c‐Myc.^114^

### 4.4 CSF1/CSF1R axis

CSF1R is the core of many diseases and is expressed at a high level on TAMs. The CSF1/CSF1R axis helps to increase recruitment and infiltration of TAMs and promote the progression and metastasis of HCC. Overexpression of solute carrier family 7 member 11 (SLC7A11) in HCC cells causes metastasis of HCC by upregulating PD‐L1 and CSF1 via the αKG/HIF1α axis, simultaneously supporting infiltration of TAMs and MDSCs in tumors via the CSF1/CSF1R axis.^40^ MiRNA is dysregulated in many types of malignant diseases. MiR‐148b deficiency induces CSF1 expression, which then binds to its receptor CSF1R to further induce macrophage infiltration into TME, thereby benefiting HCC metastasis and indicating poor prognosis.^138^ Osteopontin (OPN), a prominent tumor‐maintaining inflammatory mediator linked with tumor progression, metastasis, and immunosuppression, contributes to chemotactic migration, M2 polarization of macrophages, and PD‐L1 expression in HCC by activating the CSF1‐CSF1R axis in macrophages.^93^ In the H22 tumor‐bearing mouse model, the CSF1/CSF1R inhibitor PLX3397 diminishes macrophage recruitment and M2 polarization and cooperates with immunotherapy by reshaping the tumor immune microenvironment and enhancing infiltration of CD8^+^T cells and mature DC cells.^39^ In a word, effective blockade of CSF1/CSF1R axis is the strong assistor of immunotherapy against tumors, and the synergistic effect of both will achieve better therapeutic effect.

### 4.5 Other signaling pathways/axes

Yes‐associated protein (YAP), lncRNA, and TAM are closely related, and their individuality and interaction are significantly meaningful for tumorigenesis, metastasis, treatment, and prognosis.^139^ Nogo‐B is widely expressed in most tissues and is the only subtype of the family expressed in the liver. The tumor microenvironment assists Nogo‐B expression on macrophages to promote TAMs M2 polarization through initiating the Yap/Taz pathway, and verteporfin, an inhibitor of Yap, blocks Nogo‐B‐Yap/Taz‐mediated M2 polarization to inhibit HCC progression.^92^ High expression of YAP in human HCC cells induces hepatocytes to secrete IL‐6 recruiting macrophages to the tumor, so suppression of YAP pathway blocks macrophage chemotaxis and infiltration in HepG2 xenograft tumors.^107^ SPON2‐α4β1 integrin signaling activates RhoA and Rac1, adds F‐actin reorganization, and stimulates M1‐like macrophage recruitment to repress tumor metastasis, and at the same time, F‐actin buildup promotes YAP nuclear translocation, prevents LATS1 phosphorylation, and starts the production of downstream genes to activate the Hippo pathway.^140^ What's more, lncRNA, in close contact with TAMs, can be expressed in TAMs and affect TAMs. LncRNA H19, positively correlated with in situ CD68^+^ TAM and induced by TAMs, motivates EMT and stem cells and accelerates HCC cell invasion through triggering the miR‐193b/MAPK1 axis.^120^ Long non‐coding RNA GAS5 overexpression prevents M2‐like polarization of TAMs in SMCC‐7721 cells by regulating PTEN expression.^82^ For this reason, further research on the oncogenic mechanisms of lncRNA, YAP, and TAM and the realization of multi‐target therapy will be conducive to alleviating tumor progression and improving patient survival.

Other signaling pathways/axes, such as PI3‐K and MAPK signaling pathway,^24^, ^63^ HMGB1/TLR2/NOX2/autophagy axis^65^ and IFNG/IFR1/HHLA2 axis,^76^ also regulate TAMs to act on HCC, offering a new direction for future anti‐tumor therapy.

## 5 IMPACT OF TUMOR MICROENVIRONMENT ON TAMS IN HCC

### 5.1 Hypoxia/metabolism

Tumor‐associated macrophages are influenced by different factors in the tumor microenvironment, facilitating tumor development, and in the meantime, developing resistance to anti‐tumor therapy. First of all, hypoxia is a hallmark of the solid tumor microenvironment, and tumor hypoxia is considered a major detrimental factor in cancer treatment. The median PO_2_ of normal liver tissue is 30 mmHg, while that of liver tumor tissue is 6 mmHg,^141^ so liver cancer is one of the malignant tumors with severe hypoxia. Hypoxia‐inducible factor‐1 (HIF‐1), one of the primary mediators of the hypoxic response, can activate hypoxia‐responsive genes. High expression of HIF‐1 in the HCC microenvironment facilitates HCC cell growth and metastasis and is also connected with worse prognosis for hepatocellular carcinoma.^142^, ^143^ A study has demonstrated that under sustained and severe hypoxic conditions, M2 secretes more IL‐1β, which upregulates HIF‐1 via cyclooxygenase 2, thus leading to EMT and metastasis in HCC.^144^ Hypoxia produces HMGB1 expression in human and mouse hepatocellular tumor cells in a HIF‐1α‐dependent manner and expands macrophage infiltration and reprogramming to elevate IL‐6 secretion, subsequently promoting EMT, invasion, and metastasis of HCC cells.^32^ Moreover, TREM‐1 TAMs are abundant in hypoxic tissues of HCC, especially in advanced stage and impair CD8^+^T‐cell function, connected with poor prognosis, because TREM‐1^+^ TAMs increase chemokine (CC motif) ligand 20 expression through the extracellular signal‐regulated kinase/NF‐κβ pathway and recruit CCR6^+^Foxp3^+^Treg, causing immunosuppression.^20^ TREM‐1^+^ TAMs have higher expression of programmed cell death ligand 1 (PD‐L1) under hypoxia as well.^20^ Hypoxia can induce apoptosis, limit tumor size of HCC, and even make it disappear, but in the tumor microenvironment, it can also enhance the adaptive capacity of tumor cells in different ways toward malignant acceleration.

Hypoxia brings alterations in a range of metabolic pathways through HIF, consisting of glycolysis. In hypoxia, metabolism shifts from oxidative to glycolytic metabolism through HIF‐dependent upregulation of pyruvate dehydrogenase kinase 1 (PDK1) and lactate dehydrogenase A (LDHA).^145^ However, even under aerobic conditions, tumor cells break down glucose into lactate via glycolysis to rapidly meet the energy requirements for cell proliferation, which is called the Warburg effect. In HCC cells, lactate, a byproduct of glycolysis, augments VEGF and arginase 1 (Arg1) expression through HIF‐1α and motivates M2 polarization.^146^ An experiment in vivo has confirmed that high expression of ECT2 is a proven independent prognostic risk factor for HCC and may also promote M2 macrophage polarization by enhancing aerobic glycolysis and inhibiting immune cell function, which will cause HCC cells to proliferate and migrate.^147^ Aerobic glycolysis motivates CA12 upregulation in macrophages through HIF1α and autocrine cytokine‐dependent pathways, which not only mediates macrophage survival in a relatively acidic tumor microenvironment, but also induces macrophages to generate large amounts of CCL8, thereby facilitating EMT and the metastasis of cancer cells.^37^

In addition to glycolysis, fatty acid metabolism is another significant metabolic change in cancer cells. The dysregulated FA oxidation, sometimes referred to β‐oxidation, is another manifestation of dysregulated fatty acid metabolism in various cancers.^148^ It has been shown that FAO inhibition is associated with the development of HCC,^149^, ^150^ and yet fatty acid oxidation can supply energy to tumors, playing a certain role in pro‐tumor function of TAM. In a mimic model in vitro, FAO is responsible for the upregulation of IL‐1β secretion in a reactive oxygen species and NLRP3‐dependent manner, mediating the migration of M2 MDM and boosting the proliferation, migration, and invasion of HCC cells.^66^ Receptor‐interacting protein kinase 3 (RIPK3) is a central factor in necroptosis, and hence, RIPK3 deficiency not only decreases reactive oxygen species but also significantly inhibits caspase1‐mediated PPAR cleavage, promoting fatty acid metabolism containing fatty acid oxidation (FAO), inducing M2 polarization in the tumor microenvironment.^99^ Metabolism in the tumor microenvironment provides energy for tumor growth and also plays a role of a facilitator in TAMs‐induced tumor progression.

### 5.2 Cytokine

Cytokines are also one of the essential elements of tumor microenvironment, which can be categorized as tumor necrosis factors, interferons, interleukins, colony‐stimulating factors, and more. There are various types and different functions, all of which are irreplaceable in the development of tumor. IL‐6, closely connected with STAT3 pathway, not only upregulates miR‐25‐3p through STAT3/c‐MYC signaling and targets 3′UTR to downregulate PTPRO expression in HCC monocytes, thus promoting PD‐L1 expression in macrophages to magnify T‐cell exhaustion in HCC, but also controls PTPRO expression through IFN‐γ‐dependent mechanism.^79^ In contrast, IL‐37 hinders tumor growth by promoting the polarization of TAMs from M2 to M1 by blocking IL‐6/STAT3 signaling.^78^ Furthermore, IFN‐α suppresses the M2 phenotype of TAM induced by IL‐13, making a decline in M2 markers and a significant rise in M1 markers and neutralizing the positive function on proliferation and invasion of HCC cells, and as a result, IFN‐α acts as an adjuvant therapy to sorafenib to develop its anti‐tumor capacity.^41^ In conclusion, cytokines involved are important potential targets in the treatment of HCC, providing theoretical support for the generation of new treatment options.

### 5.3 Non‐coding RNA

Non‐coding RNAs have also been discovered to participate in the development of HCC by affecting TAMs. MicroRNA (miRNAs) are a large family of small molecule non‐coding single‐stranded RNAs, the universality and diversity of which suggest that they have many important biological functions. MiR‐98 mimics notably elevate the levels of IL‐1β and TNF‐α in TAM under hepatocellular carcinoma conditions, but significantly lower IL‐10 and TGF‐β, meaning that M2 turns into M1, thus repressing EMT, invasion, and migration of SMMC7721 and HepG2 cells.^88^ Further study has revealed that IL‐10 is the target gene of miRNA‐98, and miRNA‐98 is directly linked to the 3′UTR of IL‐10 to inhibit HCC.^87^ MiR‐144 and miR‐451a, respectively, target HGF and MIF to stimulate M1 polarization and form a negative feedback regulatory circuit with EZH2 in HCC, revealing the criticality of chromatin remodeling involved in DNA methylation‐induced gene silencing at the same time, regulating miR‐144/miR‐451a cluster expression and further controlling plasticity of TAM.^85^ Long non‐coding RNA (lncRNA) GAS5, which is also involved in TAM polarization, constricts the proliferation and invasion of SMCC‐7721 cells by enhancing the expression of PTEN to suppress M2 and promote M1.^82^

### 5.4 Exogenous drug

Current studies have discovered that some drugs originally applied in other diseases are also relevant for TAMs in HCC. Cholestyramine, a BA chelator for hyperlipidemia, reverses the polarization of M2 TAMs due to SIRT5 deficiency which demonstrates that metabolic dysregulation can cause the formation of an immunosuppressive tumor microenvironment conducive to the development of HCC.^61^ Propofol, an intravenous anesthetic, has been injected into the abdominal cavity of tumor‐bearing mice at different doses, and the tumor size is significantly reduced in a dose‐dependent manner.^95^ Low and medium doses mainly exert anti‐tumor activity, while high doses directly inhibit tumor growth.^95^ It has been further found that propofol stimulates TAMs to secrete microvesicles (MVs) that transport miR‐142‐3p into HCC cells, then inhibiting tumor cell migration.^95^ Statins, inhibitors of HMG‐CoA reductase, block the YAP pathway that stimulates IL‐6 expression, strongly impeding chemotaxis of TAMs.^107^ Genipin inactivates inosital‐requiringenzyme‐1α (IRE1α), a key factor in macrophage initiation, which declines the initiation of TAMs and secretion of pro‐inflammatory factors, significantly reduces the migration and infiltration of TAMs and effectively constrains the in situ growth of HCC in vivo.^69^ In short, more and more drugs have been testified to possess anti‐tumor function and may become candidates for tumor treatment in the future, but more research is still needed to verify in order to ensure their safety and efficacy.

## 6 SUMMARY AND FUTURE PERSPECTIVE

Tumor‐associated macrophages are gradually occupying an important place in studies on HCC. In this review, we summarize new developments in the mechanism of TAMs in HCC, as well as exploring implications of modulating TAMs in HCC drug resistance. We have demonstrated the influence of several major signaling pathways and multiple substances in the tumor microenvironment in the oncogenesis of TAMs, and at the same time, we have discussed the combination of multiple HCC therapies through modulating TAMs, which effectively mitigates drug resistance and enhances curative efficacy, with the promise of improved prognosis and better survival. There have been several reviews talking about origin, pathogenesis, or therapeutic strategies between TAM and HCC,^151^, ^152^, ^153^, ^154^, ^155^ and this paper both completes some parts of them and detailing the crosstalk between TAM and HCC from a different perspective. This article complements them by making the relationship between TAM and HCC clearer, providing a more complete understanding of TAM and HCC, and offering valuable information for the future discovery of effective strategies for targeting TAM for the treatment of HCC. As the conclusions are mainly argued on various models, whether similar results can be obtained in humans needs further confirmation.

Most studies have shown that M2 TAMs are a major factor in carcinogenesis, with higher M2 indicating high drug resistance and predicting poor prognosis, while M1 exhibits anti‐tumor effects. Therefore, in terms of therapeutic strategies, research will focus on how to inhibit M2 recruitment, reduce the number of M2, or convert M2 into M1. However, is it true that the lower the density of M2 is, the greater the anti‐tumor effect will be? Does a higher M1/M2 ratio imply worse drug resistance and better prognosis? Nowadays, there are evidences that M1 has pro‐tumor function in HCC.^156^, ^157^ The function of M1 deserves further in‐depth study. Furthermore, the M1/M2 ratio should be in relative equilibrium like Th1/Th2 in order to maintain normal physiological function, and there may be maximum and minimum thresholds of the ratio for making a difference. To sum up, functional mechanisms of TAMs still require us to explore continually to furnish a theoretical basis for future cancer treatment, and meanwhile, the strategy of combination therapy is supposed to be widely carried out to achieve more satisfactory efficacy of anti‐tumor therapy.

## AUTHOR CONTRIBUTIONS

**Xinyi Zhang:** Conceptualization (equal); investigation (lead); writing – original draft (lead). **Chao Yu:** Investigation (supporting); resources (equal). **Siqi Zhao:** Conceptualization (equal); resources (equal). **Min Wang:** Resources (equal). **Longcheng Shang:** Conceptualization (equal); supervision (equal). **Jin Zhou:** Supervision (equal); writing – review and editing (equal). **Yong Ma:** Supervision (equal); writing – review and editing (equal).

## FUNDING INFORMATION

This work was supported by the National Natural Science Foundation of China (82203762), the Chen Xiaoping foundation for the development of science and technology of Hubei Province (CXPJJH12000009‐08), and the Xinghuo Talent Program of Nanjing First Hospital.

## CONFLICT OF INTEREST STATEMENT

The authors declare that they have no competing interests.

## Data Availability

Data sharing is not applicable to this article as no new data were created or analyzed in this study.
